# Exploration of the potential mechanism of Yiyi Tongfeng Formula in the treatment of acute gouty arthritis based on network pharmacology and molecular docking: A review

**DOI:** 10.1097/MD.0000000000039609

**Published:** 2024-09-13

**Authors:** Boyang Tan, Tao Tao, Dongyang Lin, Qingyuan Yu, Fengling Sun, Zhenhua Li

**Affiliations:** a College of Integrative Medicine, Changchun University of Chinese Medicine, Changchun, Jilin, China; b College of Traditional Chinese Medicine, Changchun University of Chinese Medicine, Changchun, Jilin, China; c Affiliated Hospital of Changchun University of Chinese Medicine, Changchun, Jilin, China.

**Keywords:** acute gouty arthritis, molecular docking, network pharmacology, Yiyi Tongfeng Formula

## Abstract

The global prevalence of gout is on the rise. Yiyi Tongfeng Formula (YTF), a traditional herbal compound, has gained recognition for its efficacy in managing acute gouty arthritis (AGA). Despite its widespread use, the underlying mechanisms of YTF in AGA treatment remain largely undefined. This study employed network pharmacology and molecular docking to elucidate these mechanisms. We utilized the Traditional Chinese Medicine Systems Pharmacology Database and Analysis Platform, SymMap database, and various literature sources to identify active components and corresponding targets of YTF. Relevant AGA-associated targets were identified through the Genecards, Drugbank, Therapeutic Target Database, and Online Mendelian Inheritance in Man databases. A protein–protein interaction network was constructed to delineate interactions between YTF targets and AGA. Key ingredients and central targets were further analyzed using Cytoscape. Functional enrichment analyses, including Gene Ontology and Kyoto Encyclopedia of Genes and Genomes, were conducted via Metascape. Additionally, molecular docking studies were performed using PyMOL and AutoDock4. It was found that quercetin, kaempferol, and luteolin may be the main active components of YTF for AGA treatment. Gene Ontology enrichment analysis shows that the main biological processes involved are cellular responses to lipids, and inflammatory responses. Kyoto Encyclopedia of Genes and Genomes enrichment analysis suggests the involvement of the IL-17 signaling pathway, AGE–RAGE signaling pathway in diabetic complications, TNF signaling pathway, and so on. The findings suggest a multi-faceted therapeutic approach of YTF in treating AGA, involving multiple components, targets, biological processes, and signaling pathways. This comprehensive mechanism offers a foundation for further experimental validation.

## 1. Introduction

Gouty arthritis (GA) is a sterile inflammatory disease characterized by elevated blood uric acid levels, disturbances in purine metabolism, and the deposition of urate crystals in bones, joints, and other connective tissues.^[1–3]^ Acute gouty arthritis (AGA), the initial presentation of gout, typically emerges suddenly and is marked by severe pain. It is notorious for its high recurrence rate and the challenge it poses for complete eradication. Clinically, AGA manifests as redness, swelling, heat, and pain in the affected joints and surrounding soft tissues, often accompanied by restricted joint function. The most commonly affected site is the first metatarsophalangeal joint, although knees, fingers, and other joints may also be involved. The pain, which is often described as unbearable and slicing, tends to worsen at night. Recent trends indicate a rising incidence of gout worldwide, with prevalence rates estimated at 3.9% in the United States, approximately 0.9% to 2.5% in Europe, and between 1% and 3% in China.^[4–7]^ Traditional Western treatments, which focus on anti-inflammatory and analgesic measures along with uric acid reduction, are somewhat effective but frequently lead to adverse effects such as liver and kidney damage and gastrointestinal issues.^[8]^ In contrast, Traditional Chinese Medicine (TCM) offers distinct advantages in improving pain symptoms with fewer toxic side effects.

AGA is triggered by monosodium urate (MSU) crystals that activate local immunoglobulins, leading to rapid neutrophil recruitment. Studies show that over 95% of AGA patients experience MSU crystal attacks.^[9]^ The inflammatory nature of AGA has been extensively studied; for instance, Sheikh Fayaz Ahmad and colleagues have demonstrated clinically and experimentally that regulatory T cells (Tregs) can effectively suppress inflammation. Tregs are considered crucial in regulating the immune system to control inflammation, making them a significant target for arthritis treatment.^[10,11]^ Research indicates that maintaining a balance between Tregs and Th17 cells is vital for managing inflammatory diseases.^[11]^ Glucocorticoids, which are recommended for AGA treatment, act primarily by inhibiting NF-κB activation. This inhibition reduces the expression of pro-inflammatory factors such as TNF-α, IL-6, and IL-8.^[12]^ Furthermore, MSU crystals stimulate dendritic cells via the NF-κB signaling pathway to release Th17-polarizing cytokines. IL-17 not only activates T cells to produce IL-6 and IL-8 but also prompts macrophages to produce TNF-α and IL-1β.^[13,14]^

The 2023 Guidelines for Integrated Chinese and Western Medicine Diagnosis and Treatment of Gout and Hyperuricemia commonly characterize AGA by features of damp-heat-toxicity evidence.^[15]^ Numerous domestic and international studies have also suggested a link between intestinal flora imbalances and the development of gout.^[16–20]^ Yiyi Tongfeng Formula (YTF) is inspired by Yiyi Fuzi Baijiang San and Yuebi Jiazhu Decoction in “The Synopsis of the Golden Chamber.” As a classic and famous remedy for dispelling dampness and draining pus, Yiyi Fuzi Baijiang San is known to reduce swelling and drain pus, clear heat and remove toxins, and warm yang and pass stagnation. Yuebi Jiazhu Decoction has been validated in several studies for its effectiveness in reducing blood uric acid levels and significantly alleviating symptoms of pain and swelling in joints affected by acute gout.^[21]^ The synergistic combination of these 2 formulas is designed to dispel wind and dampness, dissolve blood stasis, and relieve pain, thus offering clinical relief to patients with acute gout. This study employs network pharmacology and molecular docking to explore the potential mechanisms by which YTF acts on AGA, aiming to enhance clinical understanding and application.

## 2. Material and methods

### 2.1. Screening of active components and targets of YTF

YTF comprises several components including Yiyiren (Coix Seed), Fuzi (Monkshood), Baijiangcao (Patriniae), Mahuang (Ephedra), Baizhu (White Atractylodes Rhizome), Shengshigao (Raw Gypsum), Shengjiang (Ginger), Dazao (Jujube), and Gancao (Liquorice). The constituents of YTF were sequentially inputted into the Traditional Chinese Medicine Systems Pharmacology (TCMSP) database and analysis platform.^[22]^ Active ingredients were selected based on an oral bioavailability of at least 30% and drug likeness of at least 0.18, followed by extraction of corresponding targets. For components not found in the TCMSP database, the SymMap database and related literature were consulted.^[23]^ The protein targets obtained were then normalized using the UniProt database.^[24]^

### 2.2. Prediction of AGA-related targets

Search terms “Acute Gouty Arthritis” and “Acute Phase of Gouty Arthritis” were used to identify AGA-related targets in the Genecards,^[25]^ Drugbank,^[26]^ Therapeutic target database,^[27]^ and OMIM databases. Targets from these databases were integrated and refined to delineate the final disease target.

### 2.3. Prediction of potential targets and PPI network construction of YTF for AGA treatment

Both drug and disease targets were input into the Evenn online website^[28]^ to identify common targets, followed by the construction of a Venn diagram. The protein–protein interaction (PPI) network was then built using the STRING database^[29]^ by entering potential targets and filtering out disconnected nodes. The top 20 core targets were selected based on degree values using Cytoscape 3.7.1 software.

### 2.4. Construction of the drug-compound–target-disease (D-C–T-D) network of YTF for AGA treatment

YTF’s components, AGA-related data, active ingredients, and common targets were imported into Cytoscape 3.7.1 to create a comprehensive drug-ingredient–target-disease network diagram. The top 20 key active ingredients were identified based on degree values.

### 2.5. GO function and KEGG pathway enrichment analysis

Potential targets of YTF for AGA treatment were analyzed for Gene Ontology (GO) functions and Kyoto Encyclopedia of Genes and Genomes (KEGG) pathways using the Metascape online platform.^[30]^ Results from these analyses were visualized using the microbiology letter platform.

### 2.6. Molecular docking

MOL2 structures of the top 5 key active ingredients were downloaded from the TCMSP database, and protein data bank structures of the top 4 target proteins were obtained from the RCSB Protein Data Bank. Molecular docking was performed using PyMOL and AutoDock4 software.

## 3. Results

### 3.1. The collection and screening of YTF active ingredients and targets

TCMSP database was utilized to identify active ingredients and corresponding targets for YTF, using oral bioavailability ≥ 30% and drug likeness ≥ 0.18 as selection criteria. Raw gypsum, not found in TCMSP, was supplemented using the SymMap database and relevant literature. A total of 200 active ingredients were identified, and after standardization using the UniProt database, 872 targets were obtained. Duplicate entries were removed, leaving 179 active ingredients corresponding to 250 unique targets.

### 3.2. Prediction of targets in AGA

Using the terms “Acute gouty arthritis” and “Acute phase of gouty arthritis,” disease-specific targets were sought across several databases: 254 from Genecard, 2456 from OMIM, 102 from DrugBank, and 1 from TTD, summing to 2813 initial targets. After removing 42 duplicates, 2771 targets remained.

### 3.3. Prediction of potential targets for YTF treatment of AGA and construction of PPI network

**Figure 1.**
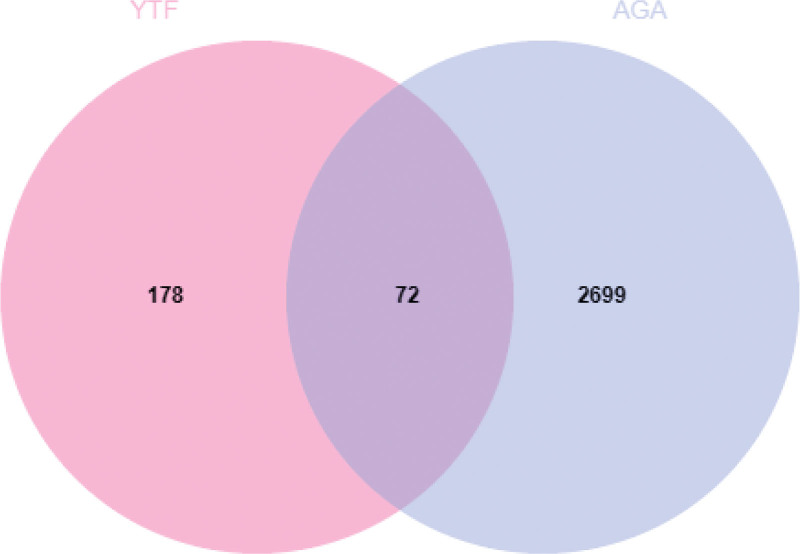
Venn diagram of common target genes for Yiyi Tongfeng Formula (YTF) and acute gouty arthritis (AGA).

**Figure 2.**
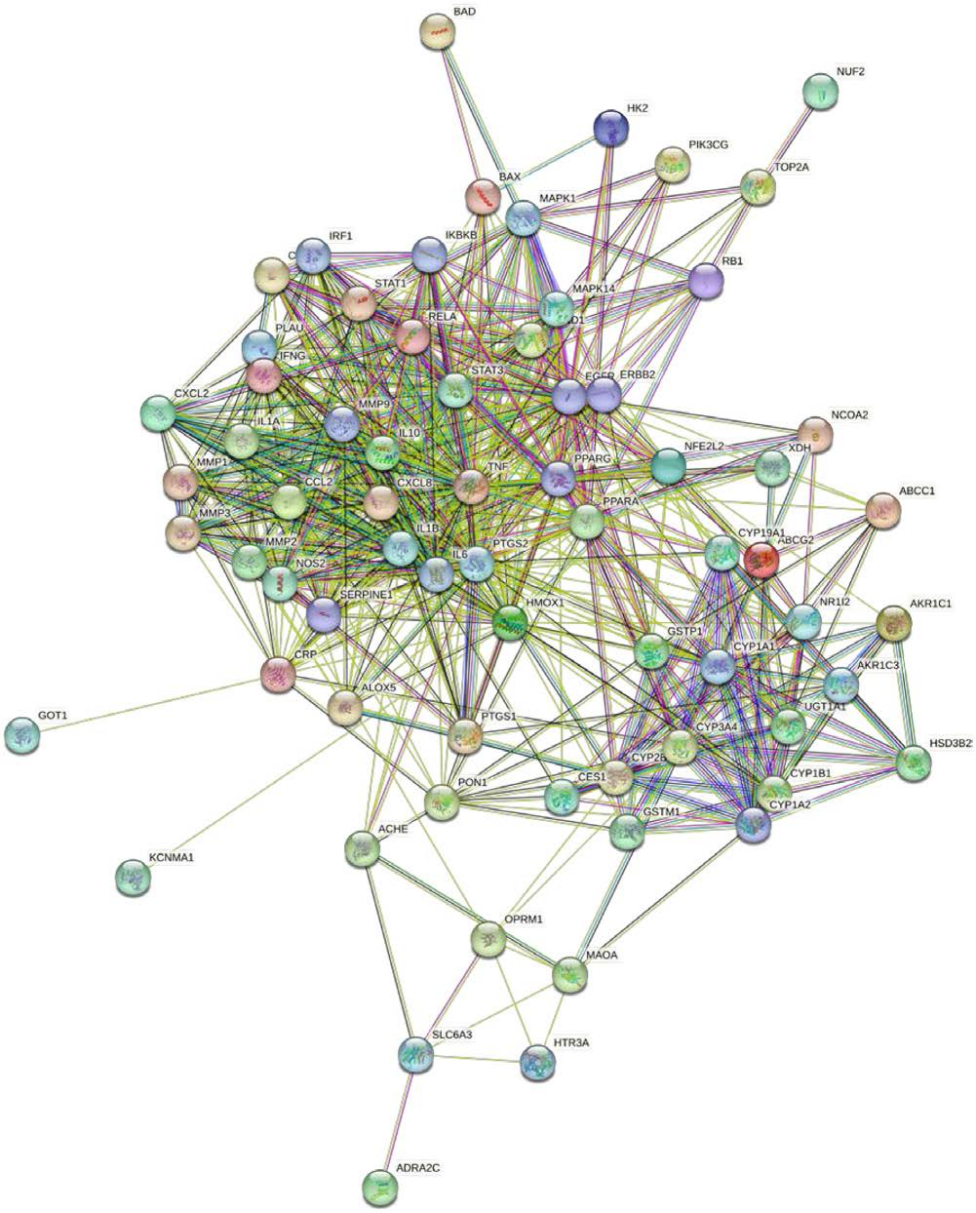
PPI network diagram from String.

**Figure 3.**
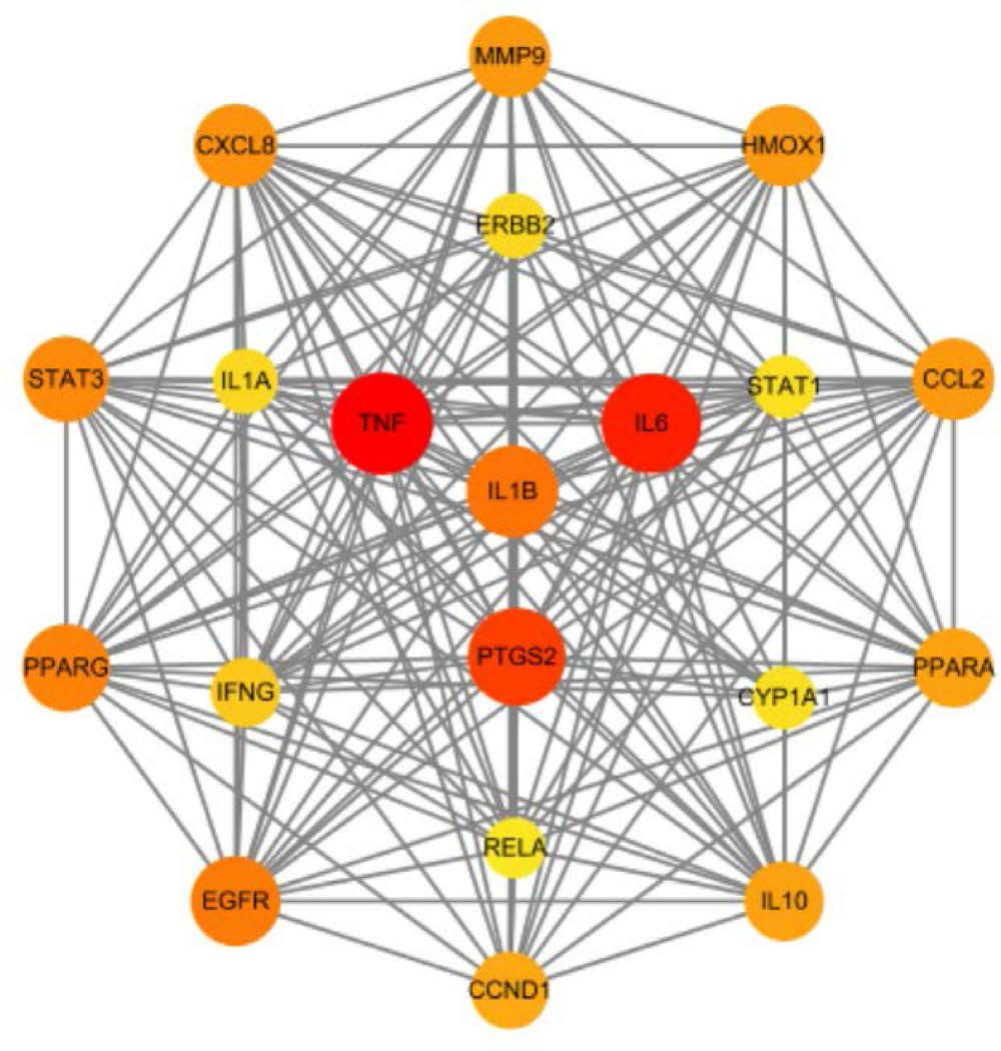
Twenty key targets determined in the PPI network.

Separate entries of drug targets and disease targets into the Evenn online tool yielded 72 potential targets for YTF in treating AGA. These were visualized in a Venn diagram (Fig. 1). The STRING database was used to construct a PPI network of potential targets for YTF treatment of AGA, resulting in a network with 69 nodes and 643 edges (Fig. 2). The top 20 core targets were identified by sorting Degree values, highlighting TNF, IL-6, PTGS2, IL-1β among others (Fig. 3). There are 20 nodes and 170 edges.

### 3.4. Construction of “drug-component–target-disease” network for AGA treatment by YTF

**Figure 4.**
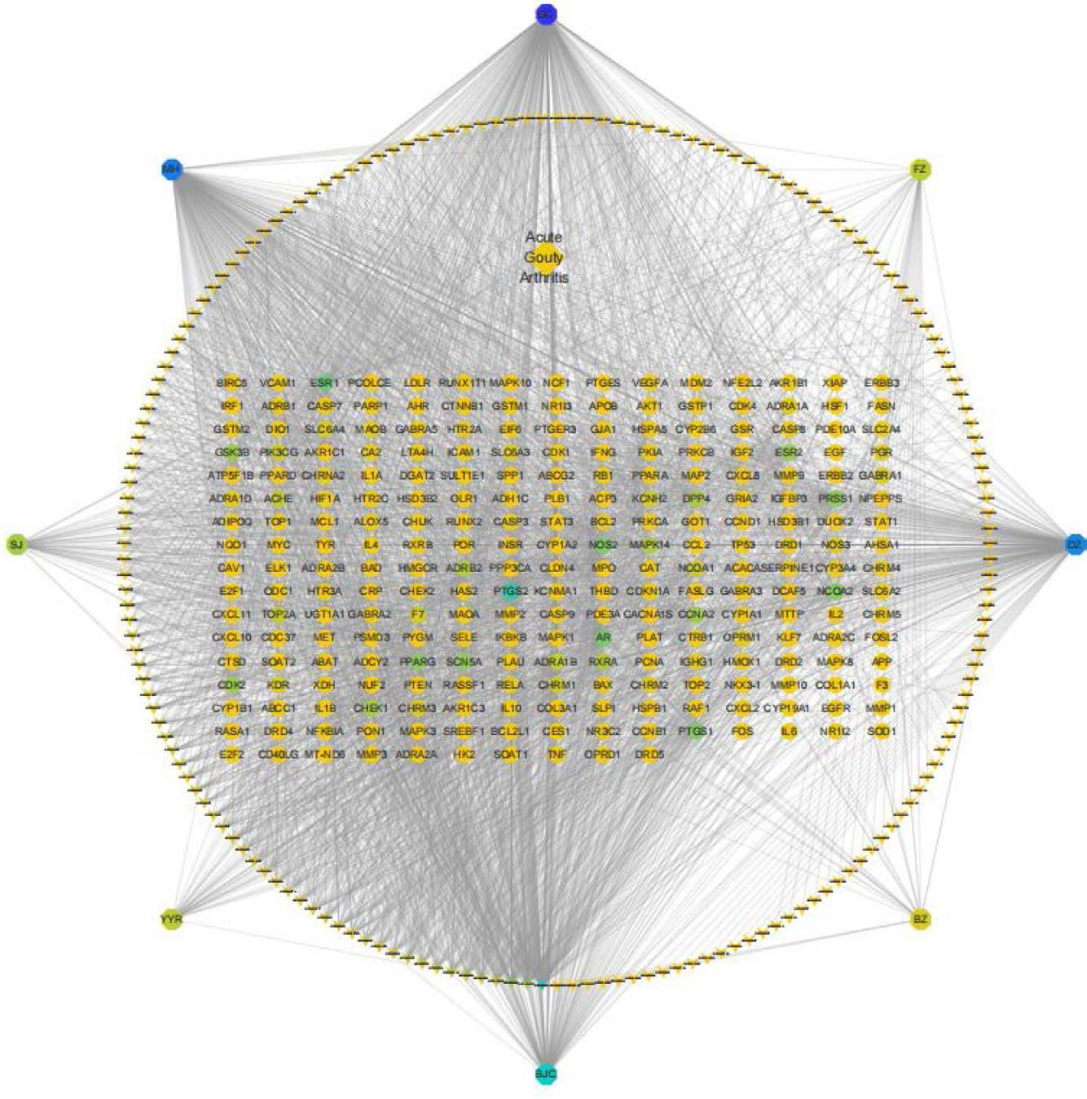
Drug-ingredient–target-disease network for YTF treatment of AGA.

Data integration into Cytoscape 3.7.1 facilitated the creation of a “drug-ingredient–target-disease” network, showcasing 437 nodes and 2984 edges (Fig. 4). The top 5 active ingredients identified by Degree value were quercetin, kaempferol, luteolin, naringenin, and Licochalcone A.

### 3.5. GO function and KEGG enrichment analysis

**Figure 5.**
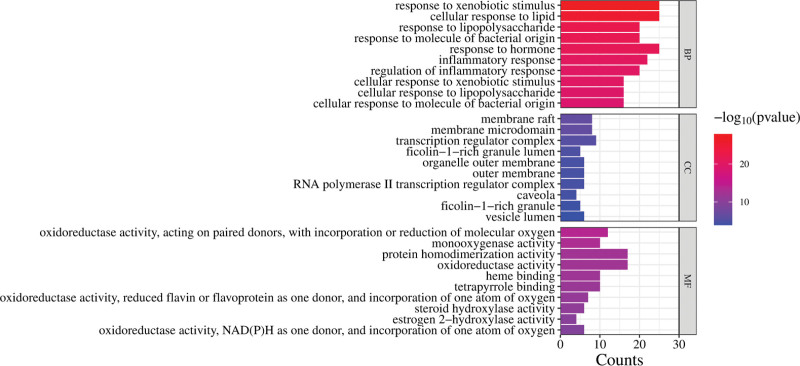
Gene Ontology (GO) enrichment analysis.

**Figure 6.**
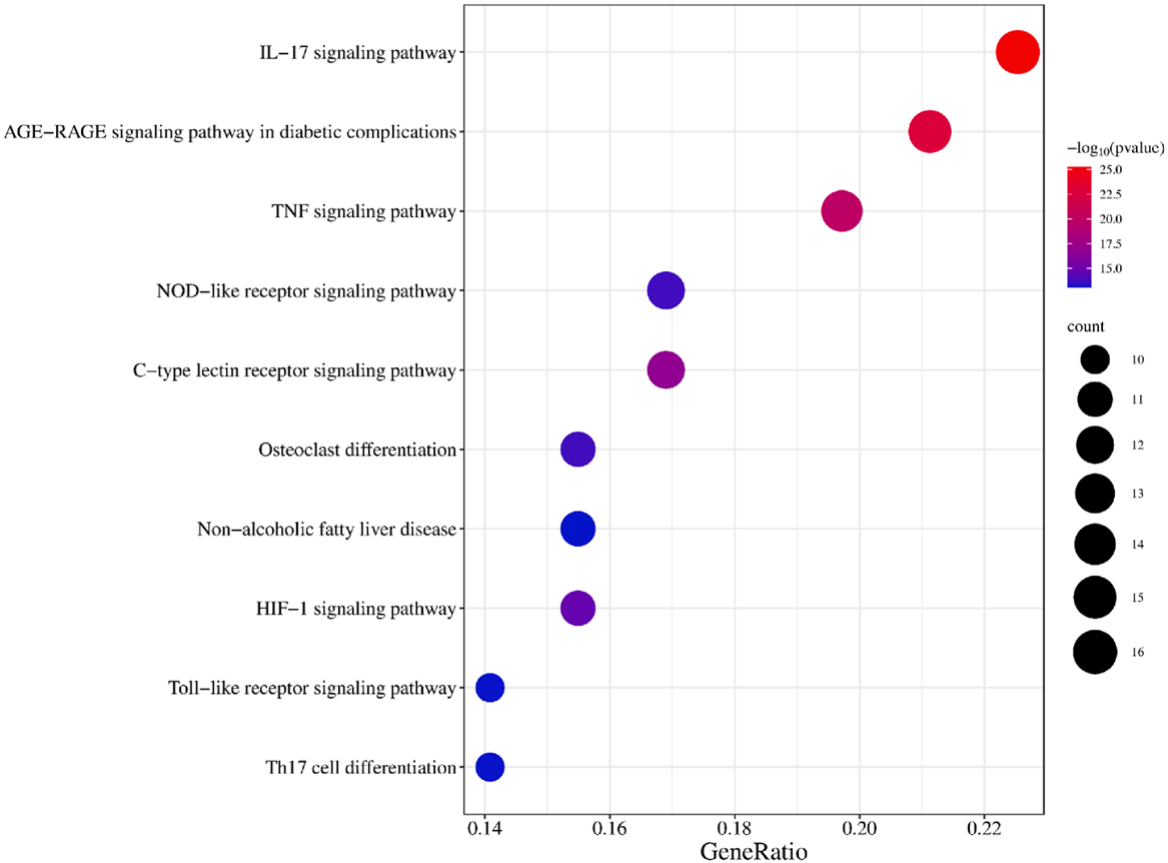
Kyoto Encyclopedia of Genes and Genomes (KEGG) enrichment analysis.

Among them, biological processes mainly include response to xenobiotic stimulus, cellular response to lipid, response to lipopolysaccharide, inflammatory response, and other related. Cellular components mainly include membrane raft, membrane microdomain, transcription regulator complex, and so on. The molecular functions are mainly related to oxidoreductase activity, protein homodimerization activity, and heme binding (Fig. 5). KEGG analysis highlighted 148 pathways, with the top 5 pathways including lipid and atherosclerosis, pathways of cancer, IL-17 signaling pathway, AGE–RAGE signaling pathway in diabetic complications, and Chagas disease. Forty-two pathways were deemed relevant to AGA. The results suggest that the pathway of YTF for AGA may be strongly associated with the IL-17 signaling pathway, AGE–RAGE signaling pathway in diabetic complications, TNF signaling pathway, and C-type lectin receptor signaling pathway (Fig. 6).

### 3.6. Molecular docking

**Figure 7.**
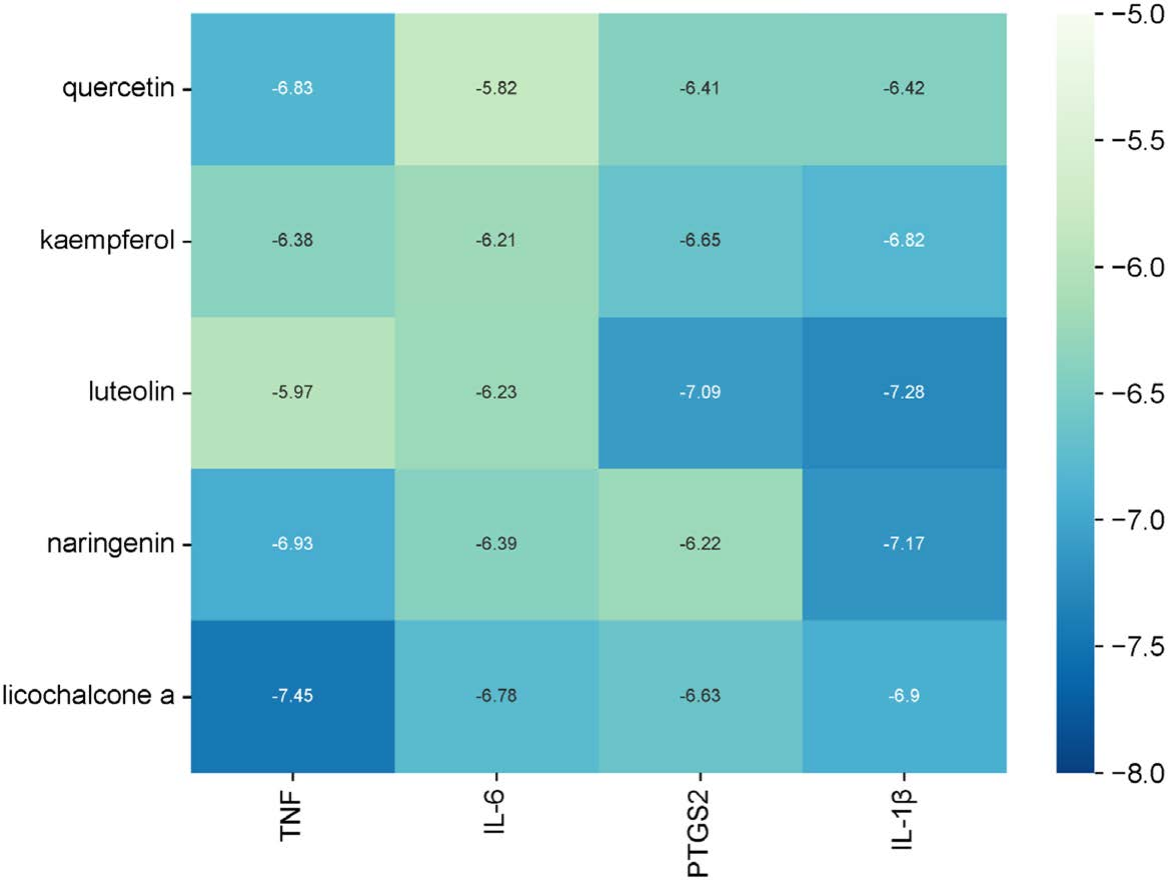
Molecular docking heat diagram.

**Figure 8.**
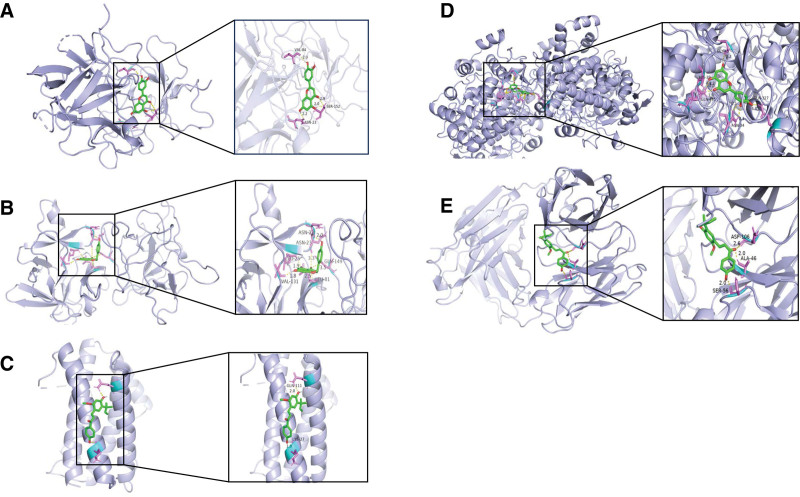
Molecular docking results of main chemical components of YTF and core proteins in PPI network. (A) IL-1B–luteolin; (B) IL-1B–naringenin; (C) IL-6–licochalcone A; (D) PTGS2–luteolin; (E) TNF–licochalcone A.

When the Binding Energy is less than −5.0 Kcal/mol, the key ingredients are considered to be in good docking status with the core target proteins. When the Binding Energy is less than −7.0 Kcal/mol, the key ingredients are considered to have strong docking ability with the core target.^[31,32]^ Molecular docking involved the top 5 active ingredients and 4 core targets, with binding energies all below −5.0 Kcal/mol indicating favorable docking (Fig. 7). Among them, luteolin showed a strong docking capacity with PTGS2 and IL-1β. Luteolin binds to specific amino acid residues of PTGS2, including GLY45, HIS39, GLN461, GLN327, and ASN34, through the formation of hydrogen bonds. It binds to the amino acid residues VAL84, SER152, and ASN23 of IL-1β by forming hydrogen bonds. Naringenin exhibits a robust binding activity to IL-1β. Naringenin binds to IL-1β by forming hydrogen bonds at protein amino acid residues ASN22, ASN23, LEU25, GLN149, LEU81, and VAL131. Licochalcone A exhibits a high affinity for TNF. It forms hydrogen bonds with amino acid residues ASP106, ALA46, and SER56 of TNF. Figure 8 displays the detailed results. Therefore, we predicted that the active ingredients of YTF could potentially treat AGA by targeting multiple core target proteins, including TNF, IL-6, PTGS2, and IL-1β.

## 4. Discussion

AGA remains one of the most prevalent forms of inflammatory arthritis globally, with its incidence and prevalence increasing annually, significantly impacting public health and contributing to societal burdens.^[33–37]^ Despite various therapeutic options, optimal management of gout remains elusive. YTF is effective in improving AGA symptoms, but its underlying mechanisms are not yet known. Thus, the study explored the potential mechanism of YTF for the treatment of AGA through network pharmacology and molecular docking techniques.

YTF, a composite formula of Yiyi Fuzi Baijiang San and Yuebi Jiazhu Decoction, combines the advantages of both and has shown remarkable efficacy in AGA treatment. Yiyi Fuzi Baijiang San is recognized for its anti-inflammatory, analgesic, antioxidant, regulation of fluid metabolism, and immunity-enhancing properties.^[38,39]^ Yuebi Jiazhu Decoction for the treatment of damp-heat AGA can not only effectively reduce the patient’s blood uric acid level, but also significantly alleviates joint pain and swelling.^[40]^ According to Professor Huang Huang, Yuebi Jiazhu Decoction is appropriate for patients who are susceptible to joint swelling and pain, particularly in the lower limbs, or have high levels of uric acid.^[41]^ In our study, 179 active ingredients of YTF were associated with 250 targets after screening through multiple databases, identifying 2771 target genes to AGA. Seventy-two potential target genes were identified for YTF treatment of AGA. Following the ranking of degree values, the main active ingredients of YTF were identified as quercetin, kaempferol, luteolin, naringenin, and licochalcone A. The active ingredients mentioned above were noted for their substantial flavonoid content, which contributes to their broad pharmacological profiles. Several studies have reported that quercetin possesses various properties, including anti-inflammatory, antioxidant, antimicrobial, anticancer, and antiaging effects.^[42,43]^ If there is an imbalance between antioxidants and oxidants in the body, it can lead to the secretion of high protein enzymes and inflammatory infiltration of neutrophils.^[44]^ Quercetin regulates both nonenzymatic and enzymatic antioxidants, including trace elements, vitamins, Reactive Oxygen Species, and glutathionase.^[44,45]^ In addition, it has strong anti-inflammatory properties and immune-modulating effects.^[46]^ Quercetin reduces inflammation by inhibiting leukocyte recruitment and decreasing levels of pro-inflammatory factors, such as IL-6 and TNF-α, as well as inflammatory mediators, such as catalase.^[47,48]^ Experimental studies have confirmed that quercetin can reduce joint edema and alleviate bone destruction and histological lesions caused by inflammation.^[49,50]^ Kaempferol has various pharmacological effects, including anti-arthritis, antioxidant, anticancer, antibacterial, and immunomodulatory properties.^[51,52]^ Due to its anti-inflammatory properties, it is commonly used to treat acute and chronic inflammatory diseases.^[53]^ Research has demonstrated that kaempferol inhibits macrophage activation, thereby reducing the production of pro-inflammatory mediators such as Thymic Stromal Lymphopoietin, IL-1β, and TNF-α.^[54]^ Animal experiments demonstrated that kaempferol effectively reduced ankle joint swelling and pain in male Sprague–Dawley (SD) rats of gout. Additionally, it significantly inhibited the expression of IL-1β, IL-6, TNF-α, and TGF-β1, resulting in anti-inflammatory effects.^[55]^ Another animal study demonstrated that kaempferol decreased serum uric acid levels and inhibited xanthine oxidoreductase activity.^[56]^ It was discovered some time ago that luteolin has greater antioxidant activity than quercetin. Additionally, luteolin’s anti-inflammatory and analgesic effects are comparable to acetylsalicylic acid.^[57]^ Naringenin reduces inflammation by decreasing the activity of mitogen-activated protein kinase and nuclear factor κB.^[58]^ Licochalcone A inhibits the expression of TLR4 and the activation of NF-κB.^[59]^ During an inflammatory response, NF-κB is highly activated and promotes the expression of inflammatory factors. Licochalcone A acts as an anti-inflammatory by inhibiting IκK and IκB.^[60]^ These active ingredients have a therapeutic effect on AGA at varying degrees.

The KEGG enrichment analysis pinpointed the IL-17 signaling pathway, AGE–RAGE signaling pathway in diabetic complications, and TNF signaling pathway as major to the action of YTF in AGA treatment. AGA mainly triggers an inflammatory response due to urate deposition in local tissues, and current in vitro studies on gout are often explored on macrophages, neutrophils.^[61]^ GA is known to activate the NF-κB and AP-1 signaling pathways, enhancing the secretion of pro-inflammatory cytokines such as IL-17, TNF-α, and IL-1β.^[61,62]^ In the IL-17 signaling pathway, IL-17 binds to the receptor and activates downstream NF-κB and MAPK pathways, resulting in increased levels of TNF-α and IL-6. Elevated levels of TNF-α and IL-6 can also increase the activity of the IL-17 inflammatory factor, thereby worsening the inflammatory response.^[63]^ IL-17 is a multifunctional cytokine with roles in the regulation of inflammation and participation in immunity and hematopoiesis.^[64]^ It plays a pivotal role in the inflammatory process, stimulating the synthesis of pro-inflammatory cytokines and prostaglandins.^[65]^ Liu et al demonstrated a statistically significant elevation in serum IL-17 levels in AGA patients 8 hours post-onset. Furthermore, they observed a gradual decline in IL-17 levels concurrent with symptomatic improvement.^[66]^ It has been demonstrated that IL-17A plays a pivotal role in neutrophil recruitment and migration, and can also stimulate osteoclast formation and bone resorption.^[65,67]^ Several studies have found that Th17 and Treg cells are closely related to the pathogenesis of GA, and the MSU-induced reduction of the Treg/Th17 ratio in the spleen of AGA rats is consistent with the development of AGA, suggesting that the potential of restoring Th17/Treg homeostasis as a therapeutic strategy to alleviating inflammation in AGA.^[62,68,69]^ The activation of Th17 cells results in the secretion of IL-17, which in turn causes the recruitment of neutrophils and accelerates the release of inflammatory factors.^[70]^ Animal experiments have confirmed that quercetin can activate the downstream pathway through the IL-17 signaling pathway, thus regulating the release of IL-6, IL-1β, and TNF-α, and alleviating gout symptoms in rats.^[49]^ To date, numerous studies have demonstrated the efficacy of IL-17 as a biomarker for the diagnosis of GA.^[71,72]^ Researchers injected MSU into transgenic mice and found that it activated the local tissue NLRP3 inflammasome at the injection site, resulting in neutrophil recruitment and elevated levels of TNF-α and IL-1β.^[73]^ There was also a positive correlation between TNF-α levels and the degree of inflammation in gout. Some researchers have proposed that modulation of inflammatory signaling may be an effective treatment for inflammatory diseases.^[74]^ Some studies have reported a positive correlation between RAGE levels and serum UA.^[63]^ Elevated UA levels are a significant factor in the development of gout. Therefore, YTF can treat AGA by inhibiting the AGE–RAGE signaling pathway in diabetic complications and TNF signaling pathways. Through the PPI network, it was discovered that the primary targets of YTF for treating AGA are TNF, IL-6, PTGS2, and IL-1β. These targets are closely associated with the KEGG-enriched pathway results. The results of molecular docking indicate that quercetin, kaempferol, and luteolin can bind highly to these core targets. This suggests that the predicted results of network pharmacology are relatively accurate. However, this study has limitations. The precise dosage of components in YTF and its impact on therapeutic outcomes were not addressed through network pharmacology. Future in vivo and in vitro studies, complemented by metabolomics and pathway validation, are essential to further elucidate the mechanisms by which YTF mitigates AGA, thereby enhancing clinical application and treatment efficacy.

## 5. Conclusion

In this study, we have systematically explored the principal active compounds in YTF for treating AGA, along with their potential mechanisms. Through network pharmacology, key compounds such as quercetin, kaempferol, luteolin, naringenin, and licochalcone A were identified. Concurrently, core targets for AGA, namely TNF, IL-6, PTGS2, and IL-1β, were delineated using PPI analyses. These targets exhibit stable binding with the major active compounds of YTF. It is posited that YTF ameliorates the clinical manifestations of AGA by modulating several signaling pathways, including IL-17, AGE–RAGE, and TNF pathways. Consequently, our findings suggest that YTF addresses AGA through a coordinated multi-component, multi-target, multi-biological process, and multi-signaling pathway approach.

## Author contributions

**Conceptualization:** Boyang Tan, Zhenhua Li.

**Data curation:** Tao tao, Qingyuan Yu.

**Formal analysis:** Boyang Tan.

**Investigation:** Tao tao, Fengling Sun.

**Methodology:** Boyang Tan.

**Supervision:** Dongyang Lin, Fengling Sun.

**Validation:** Dongyang Lin, Qingyuan Yu.

**Writing – original draft:** Boyang Tan.

**Writing – review & editing:** Zhenhua Li.
